# Selenium nanoparticle-delivered MDM2 inhibitor reactivates p53 and reprograms tumor immune microenvironment in colorectal cancer

**DOI:** 10.3389/fimmu.2025.1684611

**Published:** 2025-10-31

**Authors:** Weiming You, Jun Feng, Litao Guo, Jin Yan, Siqi Yan

**Affiliations:** ^1^ Department of Hepatology, The Second Affiliated Hospital of Xi’an Jiaotong University, Xi’an, Shaanxi, China; ^2^ Department of Tumor and Immunology in Precision Medical Institute, Western China Science and Technology Innovation Port, The Second Affiliated Hospital of Xi’an Jiaotong University, Xi’an, Shaanxi, China; ^3^ Department of Critical Care Medicine, The First Affiliated Hospital of Xi’an Jiaotong University, Xi’an, Shaanxi, China; ^4^ Institute for Stem Cell & Regenerative Medicine, The Second Affiliated Hospital of Xi’an Jiaotong University, Xi’an, Shaanxi, China

**Keywords:** selenium nanoparticles, p53 reactivation, antitumor, tumor microenvironment, peptide

## Abstract

**Background:**

Colorectal cancer (CRC) remains a major global health challenge, with limited immunotherapy efficacy in microsatellite stable (MSS) tumors that comprise ~85% of cases. The p53 tumor suppressor pathway, frequently inactivated through the mouse double minute 2 homolog (MDM2) overexpression in wild-type tumor protein 53(TP53) tumors, represents a promising therapeutic target for both direct antitumor effects and immune modulation.

**Methods:**

We developed selenium nanoparticles loaded with the MDM2-targeting peptide inhibitor MI (Se@MI) using a one-pot synthesis approach. The nanoparticles were characterized by transmission electron microscopy, dynamic light scattering, and UV-Vis spectroscopy. *In vitro* assays included MTT cytotoxicity evaluation, cellular uptake by flow cytometry, RNA-seq, and Western blotting of p53-pathway/apoptosis markers. Antitumor efficacy was evaluated in CT26 murine colorectal cancer models, with mechanistic studies including transcriptomic analysis, immunohistochemistry, and flow cytometry. Safety profiles were assessed through body weight monitoring, hematological analysis, histopathological examination of major organs, and serum biomarker evaluation.

**Results:**

Se@MI nanoparticles demonstrated uniform spherical morphology (45-50nm diameter) and displayed a positive zeta potential (+24.69 mV), indicative of favorable colloidal dispersibility. Enhanced cellular uptake (74.3% positive cells) and potent cytotoxicity (IC_50_ = 1.00 μM) were observed in CT26 cells. Transcriptomic analysis revealed significant activation of p53 signaling pathways (NES = 1.504, *P* = 0.029) and protein analyses confirmed induction of p21, PUMA, Bax, and cleaved caspase-3. *In vivo*, Se@MI treatment achieved 72.23% tumor growth inhibition, significantly outperforming controls. Mechanistically, Se@MI restored p53 function by disrupting MDM2-p53 interactions, inducing apoptosis and cell cycle arrest. Importantly, Se@MI reprogrammed the tumor immune milieu through increased infiltration of CD8+ T cell and cytotoxic function while suppressing regulatory T cells. Comprehensive safety evaluation revealed excellent biocompatibility with no adverse effects on body weight, hematological parameters, organ histology, inflammatory cytokines, or hepatic/renal function markers.

**Conclusions:**

Se@MI represents a novel nanomedicine strategy that combines direct p53 pathway reactivation with immune microenvironment modulation. This dual mechanism of action offers a potential strategy to boost immunotherapeutic outcomes in colorectal carcinoma and may help overcome resistance mechanisms to immune checkpoint blockade.

## 1 Introduction

As the third most diagnosed malignancy and second leading cause of cancer deaths globally, colorectal cancer (CRC) presents a formidable healthcare challenge. GLOBOCAN 2020 data revealed approximately 1.93 million incident cases and 940, 000 deaths globally (1, 2). The insidious nature of early-stage symptoms frequently results in late-stage diagnosis, contributing to poor patient outcomes, with epidemiological projections indicating that the CRC disease burden will surge by 60% by 2030 (3). Despite immunotherapy’s transformative impact on cancer care, CRC patients derive limited benefit, with efficacy largely restricted to the MSI-H/dMMR subset. In contrast, the majority of CRC cases (~85%) exhibit microsatellite stable (MSS) phenotypes that demonstrate limited responsiveness to immune checkpoint inhibitors (ICIs) (4, 5). This therapeutic gap necessitates the development of innovative therapeutic approaches to enhance clinical outcomes for the broader CRC patient population.

The tumor suppressor p53, widely recognized as the “guardian of the genome, “ plays a crucial role in cancer pathogenesis through its critical functions in maintaining genomic stability and regulating antitumor immune responses (6, 7). In response to cellular stress, such as DNA damage, activated p53 protein induces cell cycle arrest or apoptosis, effectively preventing malignant transformation (7, 8). Notably, TP53 mutations are found in roughly half of all CRC cases (50-60%), disrupting normal p53 tumor suppressor pathways and promoting carcinogenesis. However, nearly half of CRC patients retain wild-type TP53 status, presenting a potential therapeutic window for functional restoration strategies. Beyond its traditional cell-autonomous tumor suppressive functions, p53 serves a critical function in orchestrating antitumor immunity. Loss of p53 function significantly impairs NK cell immunosurveillance and diminishes type I interferon induction through innate immune pathways, including the cGAS/STING axis, consequently leading to immunosuppression and immune evasion (9, 10). In patients with wild-type TP53, overexpression of its negative regulator MDM2 is frequently identified as the primary mechanism underlying p53 inactivation (11). MDM2 serves as a dedicated E3 ubiquitin ligase for p53, orchestrating its degradation and transcriptional inhibition. In CRC, MDM2 overexpression drives tumor progression and therapeutic resistance while compromising tumor immunogenicity through downregulation of MHC class I expression and promotion of immune evasion (12–14). Therefore, therapeutic intervention targeting MDM2-p53 binding represents a promising therapeutic strategy to restore tumor suppressor function, reversing immunosuppressive microenvironments, and overcoming treatment resistance.

Despite the inherent challenges in targeting MDM2-p53 protein-protein interactions, multiple small molecule and peptide inhibitors have been developed, with the high-affinity peptide UPROL-10e representing a prototypical molecule with demonstrated efficacy (15). However, natural L-peptides face significant translational limitations, including rapid proteolytic degradation, short half-life, and poor cellular permeability, thereby constraining their clinical applicability. To address these pharmaceutical challenges, nanomedicine-based drug delivery systems have emerged as effective approaches for enhancing drug stability, improving pharmacokinetic profiles, and achieving tumor-specific targeting (16–22). Selenium nanoparticles (SeNPs) have attracted significant interest as drug delivery vehicles owing to their minimal toxicity, excellent biocompatibility, ability to passively accumulate in tumors via the enhanced permeability and retention (EPR) effect, and potential immunomodulatory activities that can activate both innate and adaptive immune cells (23, 24). Beyond their intrinsic anticancer properties, SeNPs serve as versatile carriers for drug and gene delivery, improving biodistribution, prolonging circulation time, and facilitating selective tumor accumulation (25, 26). Based on these considerations, this study developed a polyvinylpyrrolidone (PVP)-stabilized SeNP carrier system for efficient delivery of the MDM2-targeting peptide inhibitor MI. This innovative strategy aims to specifically restore p53 function in CRC cells, reverse the immunosuppressive microenvironment, enhance antitumor immune responses, and inhibit tumor growth, providing novel insights for improving CRC immunotherapy efficacy with significant clinical translation potential for combination therapy with immune checkpoint inhibitors.

## 2 Materials and methods

### 2.1 Materials

All types of amino acid used in this study were procured from CS Bio (Boston CA, USA). Unless otherwise stated, all additional chemicals were obtained from Sigma-Aldrich. HPLC-grade acetonitrile and water were sourced from Fisher Scientific, while selenious acid (H_2_SeO_3_) and polyvinylpyrrolidone (PVP) were purchased from Aladdin Chemicals (Shanghai, China). All reagents were used without further purification.

### 2.2 Synthesis of drug peptide

The MDM2-targeting peptide MI (sequence: TSFAEYWALLSPC) was synthesized using a CS Bio 336X automated synthesizer employing Fmoc-based solid-phase peptide synthesis procedures following established literature protocols (27–31). Peptide assembly was carried out on suitable solid-phase resins using the *in-situ* neutralization technique originally developed by Kent and colleagues. Amino acid coupling was achieved using HBTU activation in the presence of DIPEA. Upon completion of chain assembly, the peptides were cleaved from resin and side-chain deprotection was conducted with a cleavage mixture composed of 88% TFA, 5% phenol, 5% water, and 2% TIPS. Crude peptide precipitates were acquired through treatment with pre-chilled diethyl ether; these fractions were subsequently collected via centrifugation and purified to homogeneity (>95% purity) using preparative C18 column chromatography under reversed-phase HPLC conditions. Final peptide molecular weights were validated through ESI-MS.

### 2.3 Synthesis of Se@MI

The MI peptide (2 mg) was initially dissolved in 0.5 mL of 50% (v/v) ethanol to ensure complete solubilization, followed by gradual dilution with ultrapure water (resistivity: 18.2 MΩ·cm) to achieve a final volume of 3 mL, resulting in a peptide concentration of approximately 0.67 mg/mL. In a 20 mL glass beaker, selenium nanoparticles were generated *in situ* by adding 1 mL selenious acid (H_2_SeO_3_) solution containing 10 mg/mL PVP to 6 mL of 0.1 M ascorbic acid solution. The reaction mixture was evidenced by a color change from colorless to orange-red within approximately 2 minutes, indicating SeNPs formation. The MI peptide solution was then introduced dropwise to the SeNPs suspension, and stirring was continued for 30 minutes to ensure complete peptide-nanoparticle conjugation and stabilization.

### 2.4 Characterization of Se@MI

Nanoparticle morphology and size distribution were examined using transmission electron microscopy. Sample preparation involved placing 10 μL of Se@MI solution onto 300-mesh copper grid with a carbon coating and left to dry naturally. Imaging was conducted on a Talos L120C G2 TEM operated at 120 kV.

Hydrodynamic diameter measurements were obtained using Malvern Zetasizer Nano ZS. Prior to data collection, the instrument was equilibrated at 25°C with a 120-second stabilization period. Sample aliquots (1 mL) were transferred to DTS0012 disposable cuvettes and inserted into the measurement cell. Data collection comprised three consecutive measurements per sample.

Optical properties and peptide conjugation were assessed using UV-visible absorption spectroscopy on a UV-2600 spectrophotometer. Appropriately diluted sample solutions were loaded into quartz cuvettes with 10 mm optical path length, using ultrapure water as the reference. The absorbance was measured over a spectral range spanning 200 to 800 nm with a spectral resolution of 2 nm and at medium scan speed.

For drug loading, the nanoparticle dispersion was centrifuged to remove the unbound peptide (12, 000 rpm, 10 min). Then supernatant was analyzed using a C18 analytical column with a linear acetonitrile gradient (10–80%) over 20 min (flow rate:1.0 mL/min. absorbance: 214nm). The residual MI content was calculated from the peak area based on a standard MI concentration.

The release behavior of MI from Se@MI nanoparticles was investigated under reductive and non-reductive conditions. Briefly, Se@MI nanoparticles were dispersed in (pH 7.4) PBS buffer containing either 10 mM GSH or GSH-free as a control. The mixtures were maintained at 37°C with gentle shaking. At specified time points, the supernatant was collected for analysis (12, 000 rpm, 10 min). Fresh PBS (with or without GSH) was replenished to sustain sink conditions. The amount of MI released at each interval was quantified by RP-HPLC, and the cumulative release ratio was subsequently derived from the initial loading content.

### 2.5 Cell culture

CT26 cells were maintained in heat-inactivated RPMI-1640 medium with 10% (v/v) fetal bovine serum under standard conditions (37°C, 5% CO_2_, humidified atmosphere). When cells reached 80-90% confluence, they were subcultured using standard enzymatic dissociation methods.

### 2.6 MTT assays

Exponentially growing cancer cells were harvested, prepared as single-cell suspensions, and seeded into sterile 96-well microplates at densities ranging from 1×10^4^–5×10^4^ cells per well. Upon adherence, various concentrations of test compounds were applied to the wells, with blank wells and PBS-treated controls included, followed by 24-hour incubation under standard conditions. MTT solution was introduced into each well two hours before the endpoint, and cells were incubated until visible formazan precipitate formation. Following removal of the growth medium, the formazan deposits were dissolved using DMSO, and optical density readings were obtained using a microplate spectrophotometer. Percentage cell survival was determined using the formula (OD_570_ treated/OD_570_ control) × 100%. Concentration–response curves were plotted, and IC_50_ values were obtained by non-linear regression analysis.

### 2.7 Cellular uptake

CT26 cells were cultured to ~80% confluence, harvested with trypsin-EDTA, and plated in 6-well plates (2×10^5^) After overnight incubation, cells were exposed to FITC-conjugated selenium nanoparticles (Se) or selenium nanoparticles loaded with MI (Se@MI) at predetermined concentrations, while controls received PBS. Following treatment, cells were rinsed three times with pre-chilled PBS to eliminate any unbound drug, then detached using trypsin-EDTA, centrifuged (1000 rpm, 5 min), and resuspended in PBS containing 2% FBS. Flow cytometry was performed with FITC detected in the FL1 channel (488 nm excitation/530 nm emission). Uptake efficiency was quantified by the proportion of FITC-positive cells, with at least 10, 000 events recorded per sample.

### 2.8 Transcriptome analyses

CT26 cells were plated in 6-well plates at appropriate density and incubated overnight for attachment. Upon reaching optimal confluence, cells were exposed to Se@MI treatment or PBS for predetermined time periods. Total RNA was isolated using TRIzol reagent, with quality assessment by spectrophotometry (A260/A280: 1.8-2.0) and gel electrophoresis. RNA sequencing libraries were constructed following standard protocols and subjected to high-throughput sequencing. Raw sequencing data underwent quality assessment, removal of adapter sequences, and mapping to the murine reference genome. Analysis of differential gene expression was carried out using DESeq2 package, with genes showing |log_2_ fold change| ≥ 2 and p-value < 0.05 for statistical significance. For duplicate gene entries, average values were calculated from multiple probes sets mapping to the same gene. Only genes expressed in at least half of the samples in each comparison group were included in the analysis. Pathway enrichment analysis was conducted via Gene Set Enrichment Analysis (GSEA) using relevant gene set collections. Additional functional annotation and pathway analysis were performed using Gene Ontology (GO) databases.

### 2.9 WB experiment

Proteins were extracted using RIPA buffer supplemented with PMSF, and equal amounts of total protein were separated by SDS-PAGE and transferred onto PVDF membranes. After blocking with 5% BSA, membranes were incubated with specific primary antibodies overnight at 4°C and then with HRP-conjugated secondary antibodies. Protein bands were visualized by enhanced chemiluminescence (ECL) and quantified using ImageJ software.

### 2.10 *In vivo* antitumor experiments

Four-week-old female BALB/c mice were procured from GemPharmatech Co., Ltd, and conducted in strict accordance with institutional animal care guidelines and received approval from the Medical Ethics Committee of XJTU (Protocol No. 2022-1355). To establish subcutaneous xenografts, CT26 cells (1 × 10^6^) resuspended in 100 μL PBS were administered into the right flank of mice using a 27-gauge needle. Tumor development was monitored daily by palpation, with precise dimensional measurements obtained using digital calipers. Tumor volume calculations employed the standard ellipsoid formula: V = (length × width²) × 0.5. When tumors reached approximately 50–100 mm³ (typically 7 days post-inoculation), mice underwent randomization into three experimental cohorts (n = 5 per group) (1): Se@MI treatment (5 mg/kg) (2), PBS control, and (3) Se (blank selenium nanoparticles without peptide). Treatments were administered via tail vein injection according to the predetermined dosing schedule. Tumor dimensions and body weights were recorded daily throughout the study period. Animals were monitored for signs of distress or adverse effects, and humane endpoints were established according to institutional guidelines. At study termination, tumors were subsequently excised, photographed, weighed, and processed for histological analysis. Primary antibodies utilized in this experiment were as previously reported (32, 33). IHC staining was quantified using a comprehensive semi-quantitative scoring approach. Staining intensity was graded on a four-tier scale: 0 (no staining detectable), 1 (faint positive with light yellow coloration), 2 (moderate positive with brownish-yellow staining), and 3 (strong positive with deep brown coloration). The proportion of positive cells was divided into four categories: 1 (≤25%), 2 (26%–50%), 3 (51%–75%), and 4 (>75%). The overall IHC score was obtained by multiplying the staining intensity grade with the proportion score of positive cells.

### 2.11 Flow cytometric analysis

Following animal sacrifice, the tumor tissues were aseptically excised and subjected to irrigation with sterile saline to any residual contaminants. Tissues were mechanically dissociated using sterile scissors in 5 mL centrifuge tubes and subsequently enzymatically digested with digestive fluid at 37°C with orbital shaking (120 rpm) for 60 minutes. The obtained cell suspension was subjected to filtration through a 100 μm cell strainer, centrifuged at 700 × g for 5 minutes to pellet cells, and resuspended in Dulbecco’s PBS.

For CTL functional analysis, single-cell suspensions were incubated overnight (37°C, 5% CO_2_) with a cell stimulation cocktail to enhance cytokine production. Stimulated cells underwent washing by centrifugation at 700 × g for 5 minutes. After removing the supernatant, the cell pellet was resuspended and labeled with extracellular antibodies for 40 minutes: APC-Cy7 anti-CD45, FITC anti-CD3, and BV605 anti-CD8. After PBS washing, cell viability was assessed using Fixable Viability Stain 700 (10 min incubation) to identify and exclude non-viable cells. Cells were then pelleted by centrifugation and subjected to fixation using fixation buffer for 30 minutes, and permeabilized twice with permeabilization solution. Intracellular staining was performed using BV421 anti-IFN-γ and APC anti-Granzyme B antibodies for 40 minutes at ambient temperature. Finally, the stained cells were resuspended in D-PBS and analyzed by flow cytometry.

Treg identification utilized the same methodology as CTL analysis but omitted the initial stimulation procedure. Surface marker detection was performed using a four-color antibody panel: APC-Cy7 anti-CD45, FITC anti-CD3, BV510 anti-CD4, and APC anti-CD25. Intracellular FOXP3 expression was detected using PE-conjugated anti-FOXP3 antibody. All antibodies were obtained from Invitrogen.

### 2.12 Statistical analysis

All quantitative data were presented as mean ± SEM. Unless otherwise stated, statistical differences between two groups using a two-tailed Student’s t-test or one-way ANOVA involving three or more groups. * *P* < 0.05 was judged statistically significant.

## 3 Results

### 3.1 Design and synthesis of selenium nanoparticle-peptide conjugates

**Figure 1:**
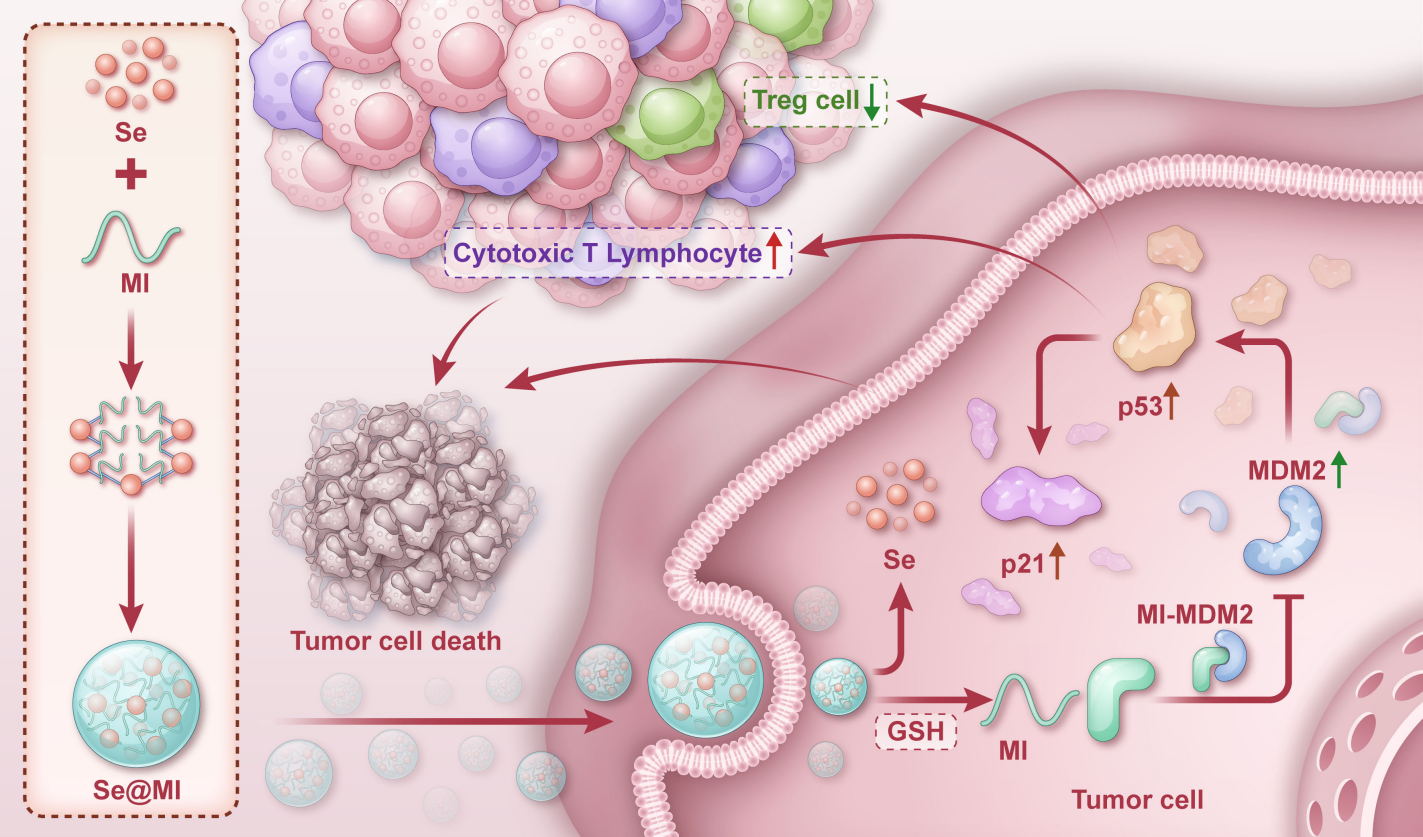
Graphical depiction of Se@MI preparation and its functional mechanism in tumor therapy. One-pot synthesis of selenium nanoparticles (SeNPs) via ascorbic acid reduction of H_2_SeO_3_, followed by MI peptide assembly through non-covalent interactions. Upon tumor cell uptake, glutathione-triggered release of MI disrupts MDM2-p53 interaction, restoring p53 function and inducing apoptosis while enhancing antitumor immunity.

**Figure 2:**
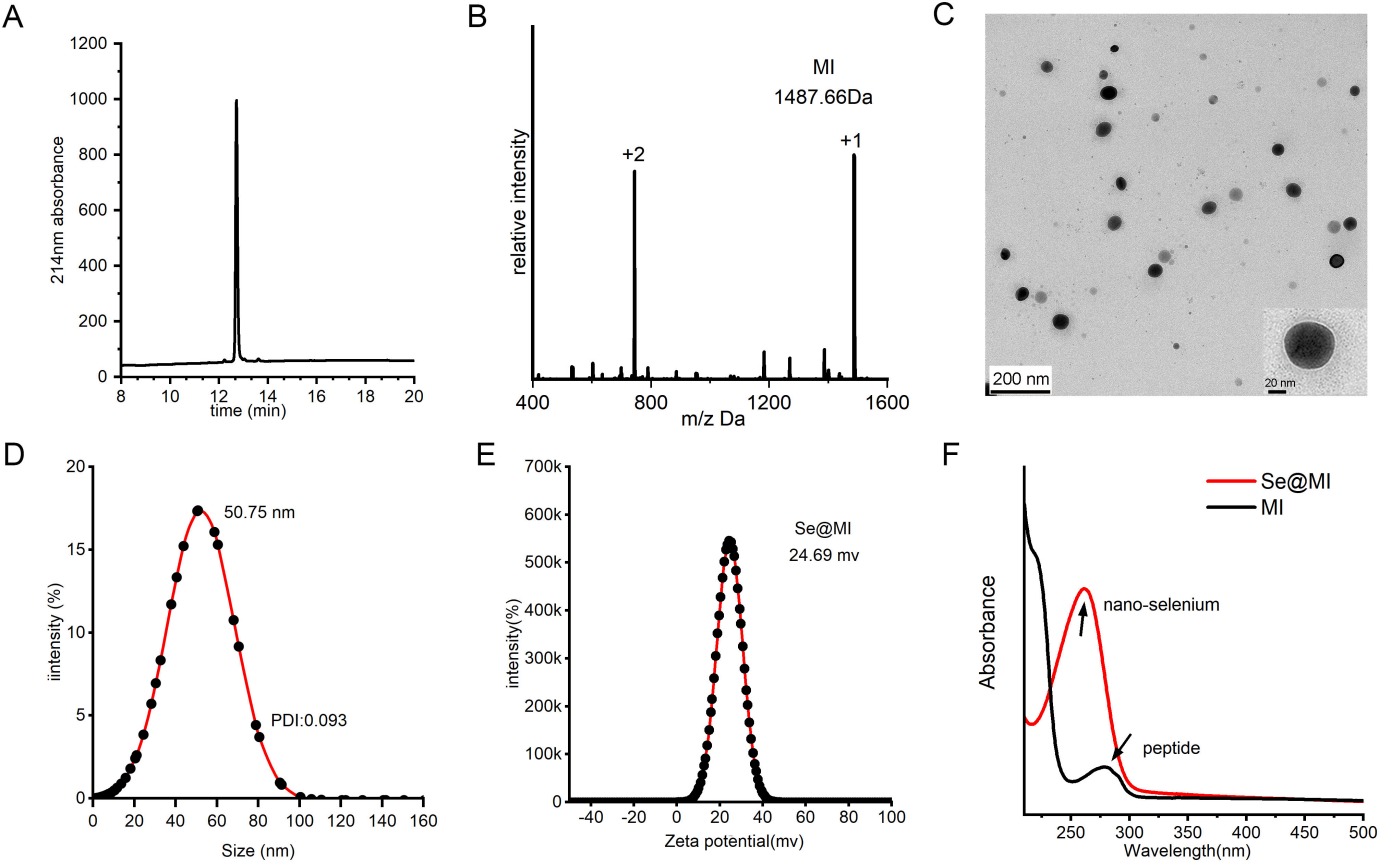
Fabrication and physicochemical profiling of Se@MI. **(A)** HPLC chromatogram of MI peptide synthesized via solid-phase chemical synthesis. **(B)** ESI-MS spectrum of MI peptide showing measured m/z values corresponding to the molecular ion species. **(C)** Transmission electron microscopy (TEM) image of Se@MI nanoparticles at 120 kV, revealing particle morphology, size, and dispersion. **(D)** Dynamic light scattering (DLS) analysis of Se@MI in aqueous solution, showing particle size distribution and average diameter. **(E)** Zeta potential analysis of Se@MI. **(F)** UV-Vis absorption spectra of free peptide and Se@MI, highlighting characteristic absorbance peaks indicating successful nanoparticle–peptide assembly.

We developed a facile one-pot synthesis strategy to create selenium nanoparticles functionalized with the MDM2-targeting peptide MI (Se@MI). The synthesis leveraged mild reduction conditions using ascorbic acid to generate SeNPs *in situ*, followed by peptide assembly through non-covalent interactions (**Figure 1**). The synthesis of peptide MI was achieved via solid-phase peptide synthesis, followed by an assessment of its purity using high- HPLC, which revealed a single major peak at approximately 10 minutes, indicative of exceptional synthetic purity (**Figure 2A**). Further validation was obtained through electrospray ionization mass spectrometry (ESI-MS), which confirmed the molecular weight as 1487 Da—aligned with the theoretical value (**Figure 2B**)—thereby substantiating the integrity of the peptide structure while negating significant side-product formation. Transmission electron microscopy (TEM) images showed that Se@MI nanoparticles exhibited a uniform spherical morphology, demonstrating excellent dispersion without any apparent aggregation. The particle diameters predominantly ranged between 45–50 nm (**Figure 2C**), with higher magnification images showcasing dense cores complemented by smooth edges typical of conventional nanostructures. DLS measurements demonstrated a mean hydrodynamic size of 50.75 nm accompanied by a unimodal distribution, pointing to commendable aqueous dispersibility (**Figure 2D**). Notably, this size range falls within the optimal window (20–100 nm) for the EPR effect, thereby promoting the preferential accumulation of nanoparticles at tumor sites owing to increased vascular permeability and dysfunctional lymphatic clearance, thereby enhancing their intratumoral accumulation and therapeutic selectivity. Furthermore, zeta potential measurements showed a surface potential of +24.69 mV for Se@MI (**Figure 2E**), suggesting strong electrostatic repulsion between particles, which contributes to colloidal stability and reduces the likelihood of aggregation during systemic circulation. Ultraviolet-visible (UV-Vis) absorption spectroscopy further validated the composite nature of the Se–peptide system: the free peptide MI exhibited characteristic absorbance between 210–230 nm, while Se@MI nanoparticles displayed additional selenium-related peaks in the 260–280 nm region, indicating successful SeNPs formation and peptide-mediated stabilization (**Figure 2F**). The successful conjugation and high drug-loading capacity of MI onto SeNPs were further validated by RP-HPLC analysis, which demonstrated a loading efficiency of 91.05% (**Supplementary Figure S1A**). Drug release studies showed that Se@MI nanoparticles exhibited GSH-responsive behavior: under reductive conditions (10 mM GSH, mimicking intracellular environments), the release of MI reached nearly 80% within 24 h, whereas under non-reductive conditions (GSH-free), less than 15% of MI was released (**Supplementary Figure S1B**). Importantly, hydrodynamic size monitoring indicated that Se@MI nanoparticles maintained excellent colloidal stability in PBS containing 20% FBS over 24 h, with negligible size fluctuations (**Supplementary Figure S1C**).

Having established the structural integrity, colloidal stability, and desirable drug-release characteristics of Se@MI, we next investigated their biological performance to assess whether these physicochemical advantages could translate into functional therapeutic benefits. To evaluate this, we first assessed their cellular uptake efficiency in CT26 colon cancer cells (**Supplementary Figure S2A**). Flow cytometry analysis revealed that Se@MI nanoparticles demonstrated significantly enhanced cellular internalization compared to controls, with 74.3% of cells showing positive fluorescence signal, while PBS treatment showed minimal uptake (0.15% positive cells) and Se alone achieved moderate uptake (33.1% positive). This enhanced cellular uptake can be attributed to the positive surface charge of Se@MI nanoparticles (+24.69 mV), which facilitates electrostatic interactions with negatively charged cell membranes, and the targeting capability of the MDM2-binding peptide MI. Subsequently, we evaluated the anticancer efficacy of Se@MI nanoparticles using MTT assay (**Supplementary Figure S2B**). The treatment exhibited potent dose-dependent cytotoxicity against CT26 cells with an IC50 value of 1.00 μM. Cell viability decreased progressively with increasing concentrations, dropping below 20% at concentrations above 4 μM. The superior cytotoxic activity observed can be directly correlated with the enhanced cellular uptake efficiency, demonstrating that the improved internalization translates to increased therapeutic efficacy. Collectively, these physicochemical characterizations and biological evaluations confirm the successful synthesis of structurally uniform, colloidally stable Se@MI nanoconjugates with dimensions and surface properties favorable for enhanced cellular uptake and potent anticancer activity, validating their potential for targeted colon cancer therapy.

### 3.2 Se@MI reactivates p53 transcriptional programs and induces apoptosis

**Figure 3:**
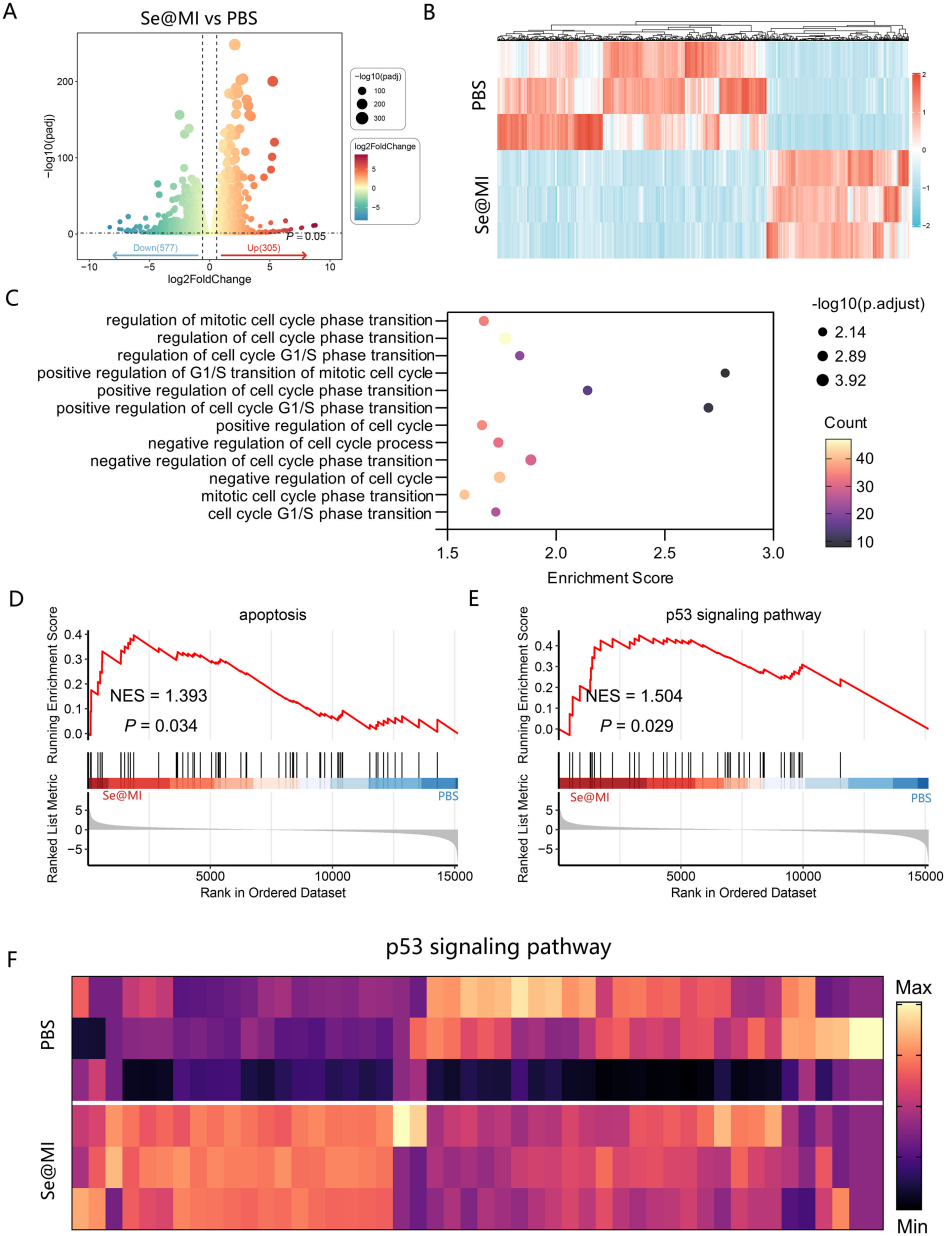
Transcriptomic analysis of Se@MI-induced p53 activation and apoptosis in CT26 cells. **(A)** Volcano plot displaying differentially increased and downregulated genes in Se@MI-treated versus control cells (Se@MI vs PBS). **(B)** Heatmap of differentially expressed genes highlighting global transcriptomic differences between the Se@MI and PBS groups. **(C)** Gene Ontology (GO) enrichment bubble plot showing biological processes associated with apoptosis **(D)** GSEA plot showing enrichment of apoptosis-related pathways for Se@MI vs PBS. **(E, F)** GSEA enrichment plot of the p53 signaling pathway and heatmap of core genes involved in the p53 cascade showing the expression levels of key genes within the pathway for Se@MI vs PBS.

To elucidate the molecular mechanisms of Se@MI action, we conducted comprehensive whole-transcriptome sequencing on Se@MI-treated and control cells. Analysis via volcano plots unveiled a substantial alteration in gene expression profiles post-Se@MI treatment, with an impressive upregulation of 306 genes juxtaposed against 577 downregulated genes (**Figure 3A**), indicating a broad transcriptional response induced by the treatment. A heatmap of differentially expressed genes further demonstrated distinct clustering between the two groups, reflecting marked transcriptomic changes in the Se@MI-treated group (**Figure 3B**). Gene Ontology (GO) enrichment analysis elucidated that these d these genes were predominantly involved in pathways governing cell cycle progression—particularly those involved in both positive and negative modulation of G1/S phase transition and broader aspects of cell cycle progression (**Figure 3C**), suggesting that Se@MI may inhibit cell proliferation through interference with critical cell cycle dynamics. Significantly, gene set enrichment analysis (GSEA) uncovered notable enrichment within both apoptosis-related pathways (NES = 1.393, *P* = 0.034) and the p53 signaling pathway (NES = 1.503, *P* = 0.029) among cells treated with Se@MI-treated group (**Figures 3D, E**). These results imply that not only does Se@MI augment p53 signaling output, but it may concurrently stimulate programmed cell death via activation of its classical pro-apoptotic targets. To authenticate these findings, we constructed a heatmap spotlighting key genes integral to the p53 signaling cascade within the Se@MI treatment group (**Figure 3F**), thereby reinforcing evidence for transcriptional activation of the p53 signaling cascade and its crucial role in initiating apoptosis. Given that Se@MI functions by blocking the MDM2–p53 interaction and restoring p53 transcriptional activity, the associated downstream gene reprogramming and apoptotic signaling may represent a key molecular mechanism underlying its antitumor effect. To confirm the transcriptomic results at the protein level, we examined the expression of p53 downstream effectors by Western blot. Se@MI treatment markedly upregulated p21, PUMA, Bax and cleaved caspase-3 compared with control groups, reflecting enhanced activation of p53-driven apoptosis pathways (**Supplementary Figure S3**). Collectively, these results indicate that Se@MI efficiently reactivates p53 transcriptional programs, leading to robust apoptotic signaling and tumor suppression.

### 3.3 Se@MI effectively inhibits CT26 tumor progression *in vivo*

**Figure 4:**
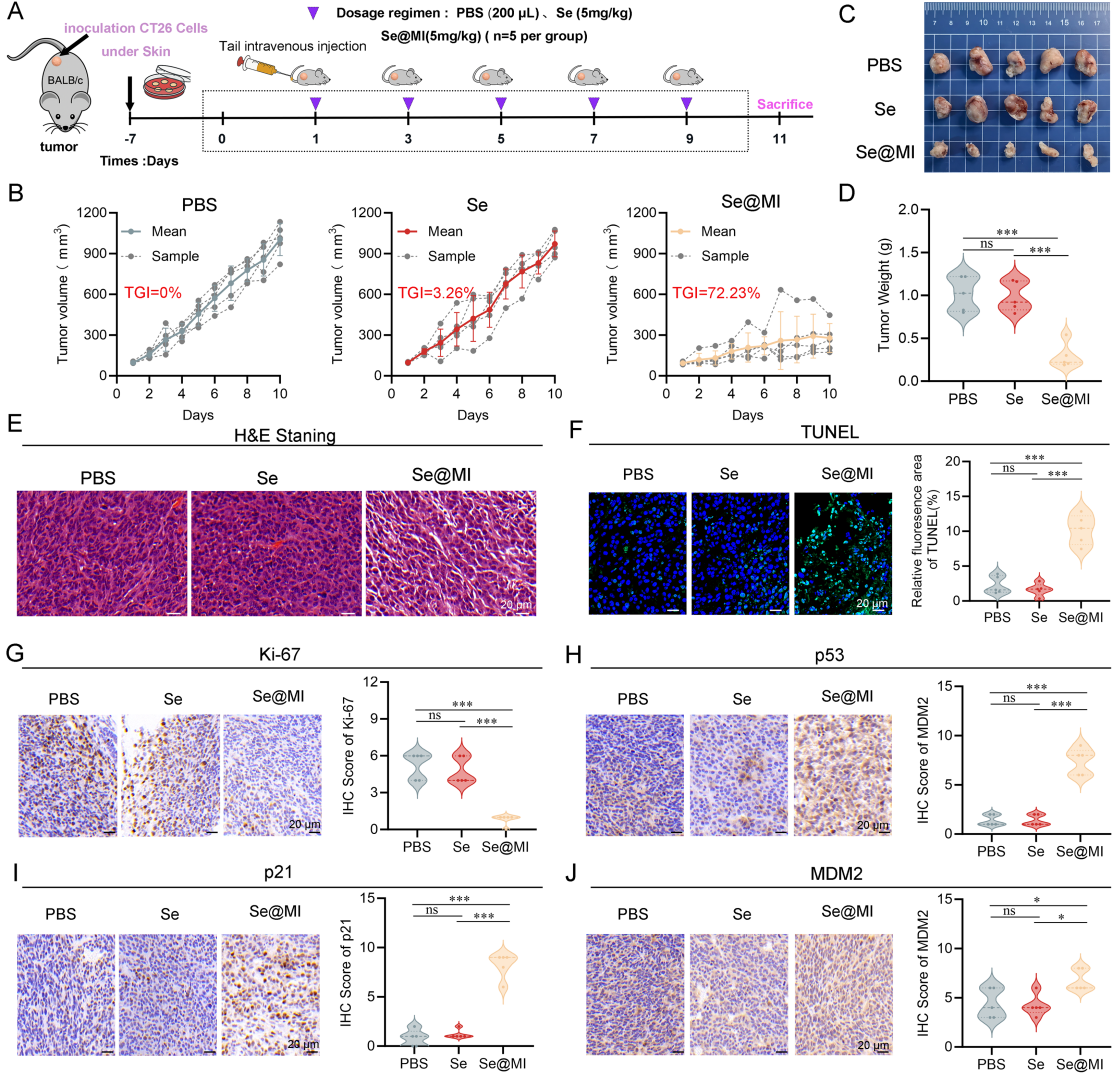
*In vivo* evaluation of Se@MI treatment in CT26 subcutaneous tumor-bearing mice. **(A)** Schematic diagram showing the treatment protocol for CT26 subcutaneous tumor-bearing mice, detailing the dosing schedule and routes of administration for each treatment group. **(B)** Tumor volume curves throughout the treatment period for each experimental group, comparing growth trends. **(C)** Representative photographs of excised tumors collected at the study endpoint from each group, showing differences in tumor size. **(D)** Tumor weights measured at the endpoint and presented for statistical comparison among groups. **(E, F)** Representative histological images of tumor sections stained with hematoxylin and eosin (HE) and TUNEL to visualize tissue morphology and apoptotic features (magnification: 40×, scale bar: 20 μm). **(G–J)** Immunohistochemical staining and corresponding IHC scores for Ki67, p53, p21 and MDM2 expression within tumor sections, depicting protein expression patterns associated with p53 signaling and proliferative activity (magnification: 40×, scale bar: 20 μm). ns denotes not statistically significant, *P < 0.05; **P < 0.01; ***P < 0.001.

To rigorously assess the antitumor efficacy of Se@MI *in vivo*, we orchestrated a subcutaneous syngeneic CT26 tumor model in BALB/c mice and meticulously randomized the subjects into three distinct groups: PBS (vehicle control), Se (blank selenium nanoparticles), and Se@MI (selenium nanoparticles loaded with the MI peptide). Adhering to a well-defined treatment regimen (**Figure 4A**), tumor volume was continuously monitored over time. Mice subjected to PBS group manifested rapid and unrelenting tumor proliferation, whereas those in the Se cohort showed tumor progression comparable to the PBS group, indicating minimal inhibitory effect. In striking contrast, the Se@MI group revealed significantly curtailed tumor expansion (**Figure 4B**), achieving an impressive tumor growth inhibition rate (TGI) of 72.23%, markedly surpassing that observed in both control groups. Photographic documentation of excised tumors at the study endpoint further provided additional corroboration for these observations (**Figure 4C**), and statistical analysis confirmed that tumor weights within the Se@MI-treated group were significantly diminished compared to both PBS and Se cohorts (**Figure 4D**). H&E staining showed disrupted tissue architecture with reduced cellularity in Se@MI-treated tumors compared to PBS and Se groups (**Figure 4E**). TUNEL staining elucidated pronounced apoptotic signaling in tumor tissues from the Se@MI group, showcasing a markedly elevated level of apoptosis relative to its counterparts (**Figure 4F**). Moreover, immunohistochemical analysis indicated considerable enhancement in expression levels of p53 alongside its downstream cell cycle inhibitor p21 within tumors derived from the Se@MI group, implying that Se@MI adeptly reinstates p53 functionality while inducing cell cycle arrest. The proportion of Ki67-positive cells was substantially among those treated with Se@MI, signifying inhibited proliferation of malignant cells (**Figures 4G–I**). Furthermore, MDM2 expression demonstrated a significant upregulation in tumors treated with Se@MI compared to both PBS and Se control groups. Immunohistochemical analysis revealed substantially increased MDM2 immunoreactivity in the Se@MI treatment group, indicating enhanced MDM2 protein levels. This elevated MDM2 expression suggests that Se@MI treatment activates compensatory cellular responses, potentially as consequence of p53 pathway activation, where increased MDM2 serves as a feedback mechanism following p53 stabilization and transcriptional activity (**Figure 4J**). Collectively, these results derived from the CT26 subcutaneous tumor model compellingly illustrate that Se@MI wields formidable antitumor efficacy *in vivo* by invigorating the p53 signaling cascade. This mechanism entails an upregulation of MDM2 expression, an enhancement of tumor cell apoptosis, and a marked suppression of cellular proliferation. Such findings accentuate Se@MI’s promise as an innovative therapeutic strategy for p53 reactivation in cancer treatment.

### 3.4 Se@MI reshapes the tumor immune landscape by enhancing CTL infiltration and suppressing Treg populations

**Figure 5:**
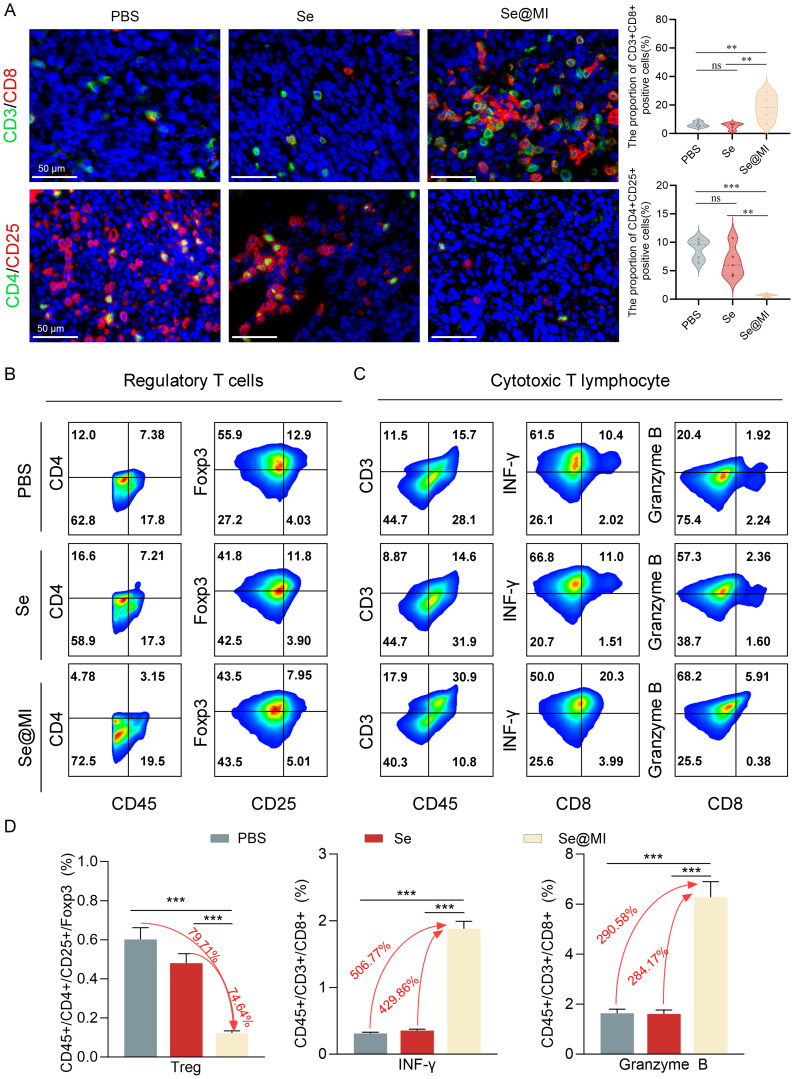
Comprehensive assessment of tumor immune microenvironment changes in CT26 tumors following Se@MI therapeutic intervention. **(A)** Dual-label fluorescence microscopy of CD3/CD8 (cytotoxic T cell identifiers) and CD4/CD25 (Treg cell markers), along with quantitative analysis of fluorescence intensity for assessing immune cell infiltration (magnification: 63×, scale bar: 50 μm). **(B)** Multi-parameter cytometric analysis of Treg populations within single-cell suspensions isolated from tumor tissues. **(C)** Flow cytometry plots analysis of Granzyme B and IFN-γ expression in CD8^+^ cytotoxic T cells. Cells were gated sequentially on CD3^+^CD45^+^ populations, and the expression levels of effector molecules were quantified. **(D)** Statistical analysis of immune cell subset proportions based on flow cytometry data, revealing differences in immune microenvironment composition between therapeutic cohorts. ns denotes not statistically significant, **P < 0.01; ***P < 0.001.

Previous studies have established that, in addition to its well-known functions in inducing apoptosis and cell cycle arrest, p53 can also modulate the TME via multiple regulatory pathways. These include enhancing antigen presentation, promoting effector T cell infiltration, and suppressing immunosuppressive factors (34, 35). In order to investigate whether Se@MI could alter the tumor immune status while activating p53, we performed immunohistochemical and flow cytometric analyses on CT26 tumor tissues. As expected, immunohistochemistry demonstrated a notable increase in CD3^+^CD8^+^ double-positive T lymphocytes within the Se@MI-treated group, indicating an enhanced infiltration of CTLs into the tumor milieu. Conversely, a substantial decrease was observed in CD4^+^CD25^+^ regulatory T cells (Tregs) compared to both the PBS and Se groups (**Figure 5A**). This suggests that Se@MI may alleviate immunosuppressive pressure and promote immune reactivation. To further assess T cell functionality, we isolated tumor-infiltrating lymphocytes (TILs) for flow cytometric analysis. Results demonstrated that CD8^+^ T cells from Se@MI group exhibited significantly elevated expression levels of Granzyme B and interferon-γ, reflecting increased recruitment as well as enhanced cytolytic activity. Notably, the proportion of Foxp3^+^CD4^+^ Tregs was markedly decreased in the Se@MI-treated group, further supporting the notion that Se@MI reshapes the tumor immune microenvironment by mitigating immune suppression (**Figures 5B–D**). Together, these findings indicate that Se@MI, through p53 pathway activation, not only triggers direct tumor cell apoptosis but also reprograms the immune contexture by enhancing CTL infiltration and activity while suppressing Treg-mediated immunosuppressive responses, thereby exhibiting dual mechanisms of tumor suppression involving both intrinsic cellular effects and modulation of immune regulation.

### 3.5 *In vivo* safety evaluation of Se@MI nanoparticles

**Figure 6:**
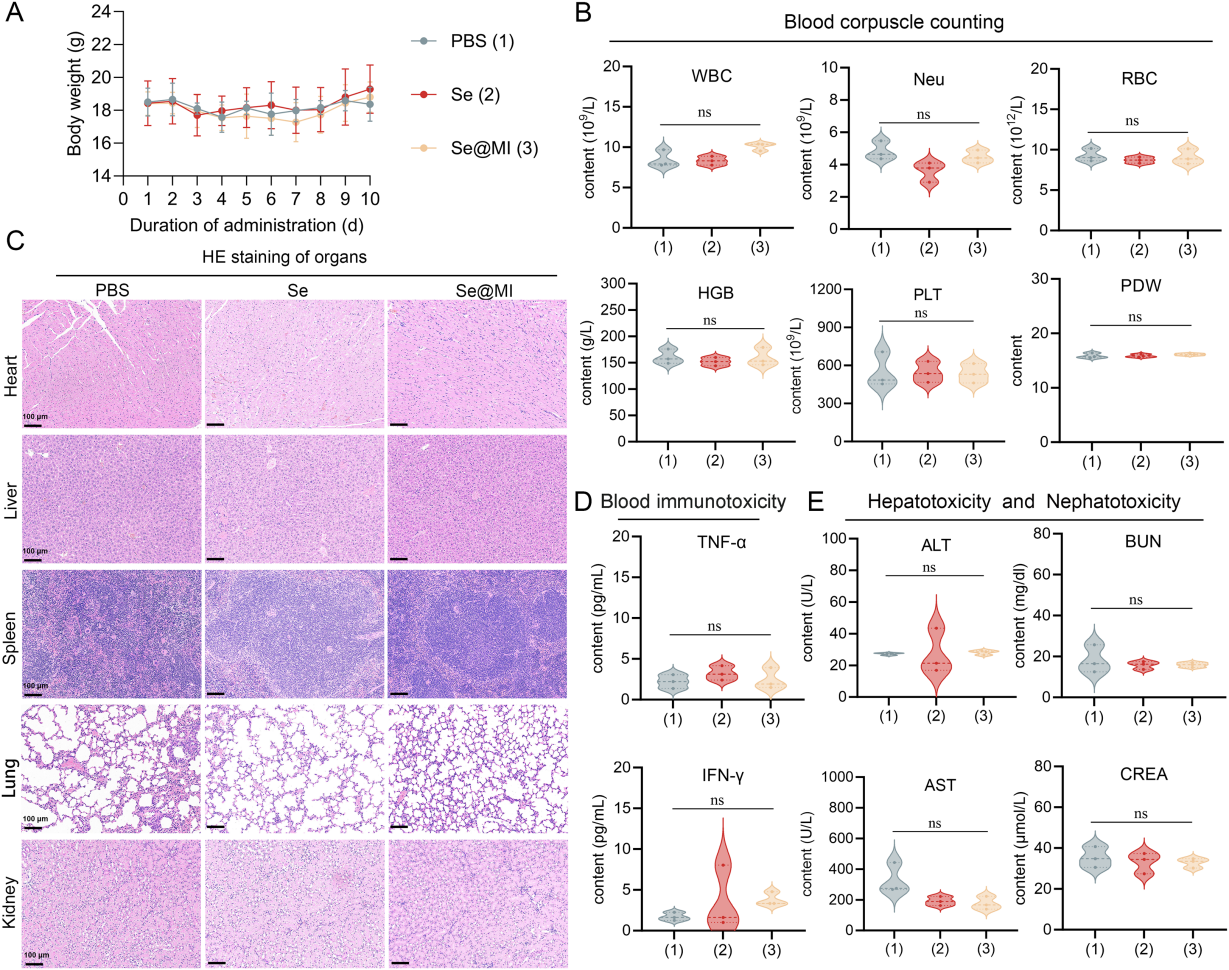
*In vivo* biocompatibility assessment of Se@MI nanoparticles. **(A)** Body weight changes of mice over 10 days following intravenous administration of PBS (control), Se, and Se@MI (n = 5/group). **(B)** Hematological analysis comparing blood cell parameters across treatment groups. **(C)** Histopathological examination of major organs via H&E staining from respective treatment cohorts (magnification: 20×, scale bar = 100 μm). **(D)** Serum levels of inflammatory cytokines TNF-α and IFN-γ in serum. **(E)** Assessment of hepatotoxicity and nephrotoxicity through serum biomarkers including alanine aminotransferase (ALT), aspartate aminotransferase (AST), blood urea nitrogen (BUN), and creatinine (CREA). Data are presented as mean ± SD. ns, not significant.

The clinical translation of nanoparticle-based therapeutics critically depends on rigorous safety evaluation, as many otherwise promising nanomedicines have failed in clinical trials due to unforeseen toxicity. Given selenium’s narrow therapeutic window and the complex biological interactions inherent to peptide-loaded nano-formulations, establishing a robust safety profile for Se@MI nanoparticles is essential for advancing toward clinical application. To this end, comprehensive *in vivo* biocompatibility studies were performed in healthy mice. Body weight monitoring over a 10-day observation period revealed no significant differences between treatment groups and the PBS control, with all animals maintaining stable body weights, suggesting excellent systemic tolerance (**Figure 6A**). Hematological analysis further confirmed the biocompatibility of Se@MI nanoparticles: complete blood count parameters showed no clinically relevant alterations in white blood cell subpopulations, red blood cell indices, or platelet parameters, all remaining within normal physiological ranges (**Figure 6B**). Histopathological examination of major organs (heart, liver, spleen, lung, and kidney) using H&E staining revealed no morphological abnormalities or tissue damage in nanoparticle-treated mice, with tissue architecture and cellular morphology comparable to PBS controls (**Figure 6C**). Systemic inflammatory responses were also evaluated, and no significant increases in pro-inflammatory cytokines TNF-α or IFN-γ were observed, indicating minimal immunogenicity (**Figure 6D**). In addition, organ function biomarkers remained unaffected: hepatic enzymes (ALT, AST) and renal indicators (BUN, CREA) showed no statistically significant differences compared to controls (**Figure 6E**). Together, these results demonstrate that Se@MI exhibits excellent biocompatibility with negligible systemic or organ-specific toxicity, supporting their potential for safe clinical translation.

## 4 Discussion

The development of effective therapeutic strategies for microsatellite stable colorectal cancer remains a critical unmet need, as these tumors constitute the majority of CRC cases yet respond poorly to current immunotherapy approaches. Our study presents a novel nanomedicine platform, Se@MI, that addresses this challenge through a dual mechanism targeting both intrinsic tumor suppressor pathways and the immunosuppressive microenvironment. The successful synthesis and characterization of Se@MI nanoparticles represent a significant advance in peptide drug delivery. The optimal size range of 45–50 nm achieved through our one-pot synthesis method is particularly noteworthy, as it falls within the ideal window for exploiting the EPR effect in solid tumors. By utilizing selenium nanoparticles as carriers, we not only protected the MI peptide from proteolytic degradation but also potentially leveraged the intrinsic immunomodulatory properties of selenium itself.

Our transcriptomic analysis provides compelling evidence for the molecular mechanism underlying Se@MI’s antitumor activity. The significant enrichment of p53 signaling pathways and transcriptional upregulation of critical pro-apoptotic mediators, demonstrate successful restoration of p53 transcriptional activity. This is particularly noteworthy given that approximately 50% of CRC patients retain wild-type TP53, making them potential candidates for p53 reactivation therapy. Furthermore, the observed signatures of cell cycle arrest provide additional support for the well-established tumor-suppressive roles associated with restored p53 activity.

The *in vivo* efficacy of Se@MI, achieving 72.23% tumor growth inhibition, substantially exceeded our initial expectations and highlights the potential clinical relevance of this approach. The concordance between reduced MDM2 expression and increased p53/p21 levels in tumor tissues validates our proposed mechanism of action—that Se@MI effectively disrupts MDM2-p53 interactions in the complex tumor microenvironment. The extensive apoptosis observed through TUNEL staining corroborates our transcriptomic findings and demonstrates successful translation of molecular mechanisms to therapeutic outcomes. Perhaps most intriguingly, our study reveals an unexpected dimension of p53 reactivation therapy—its substantial influence in reshaping the TME. The enhanced infiltration and activation of CD8+ cytotoxic T lymphocytes, coupled with reduced regulatory T cell populations, suggests that p53 restoration extends beyond cell-autonomous tumor suppression to include immune surveillance enhancement. This finding aligns with emerging evidence that p53 plays crucial roles in antigen presentation and immune cell recruitment (36–39). The elevated Granzyme B and IFN-γ levels in intratumoral CD8^+^ T cells within the tumor microenvironment indicates not just numerical increases but functional enhancement of antitumor immunity.

These immunomodulatory effects have significant implications for combination therapy strategies. Given that immune checkpoint inhibitors show limited efficacy in MSS CRC due to low immunogenicity and poor T cell infiltration, Se@MI has the potential to transform immunologically “cold” tumors into “hot” ones, thereby enhancing their responsiveness to immunotherapy. This dual actions of tumoricidal activity and immune stimulation may exert a synergistic therapeutic effect when used in conjunction with anti-PD-1/PD-L1 immune checkpoint inhibitors, potentially overcoming primary resistance to immunotherapy in MSS CRC patients.

Several limitations merit consideration. First, while our murine model provides proof-of-concept, the translation to human CRC will require careful evaluation of species-specific differences in p53 regulation and immune responses. Second, the long-term stability and potential accumulation of selenium nanoparticles warrant further investigation, although selenium’s status as an essential trace element with established safety profiles is encouraging. Relatedly, the challenges of selenium metabolism and tissue distribution, particularly the possibility of long-term accumulation and delayed toxicity, must be acknowledged and addressed in future safety studies. Third, patient stratification based on TP53 status and MDM2 expression levels will be crucial for identifying optimal candidates for this therapy; however, TP53 status alone may be insufficient, and composite molecular biomarkers or immunological profiles may be required to guide clinical decision-making. Moreover, additional validation in more clinically relevant CRC models, such as patient-derived xenografts (PDX) and patient-derived organoids (PDO), will be important to strengthen translational relevance. Practical considerations also extend to manufacturing, where scaling up production of Se@MI under GMP-compliant conditions and ensuring batch-to-batch reproducibility will be critical for advancing toward clinical trials. Looking ahead, several avenues merit exploration to further advance this work. Optimization of the peptide sequence through structure–activity relationship studies could enhance potency and specificity. Investigation of combination regimens with immune checkpoint inhibitors in immunocompetent models would provide critical insights into synergistic potential. Additionally, the development of predictive biomarkers for treatment response—potentially including circulating tumor DNA analysis of TP53 mutations, MDM2 expression levels, or immune signatures—will be essential for patient selection and real-time monitoring. Collectively, these efforts will help address current challenges and accelerate the clinical translation of Se@MI.

In conclusion, Se@MI represents an innovative therapeutic strategy that addresses fundamental challenges in CRC treatment through simultaneous targeting of tumor suppressor pathways and immune evasion mechanisms. By restoring p53 function and reprogramming the tumor microenvironment, this approach offers new hope for improving outcomes in MSS CRC patients who currently have limited therapeutic options. The successful integration of nanotechnology, peptide therapeutics, and cancer immunology principles demonstrated here provides a paradigm for developing next-generation cancer therapies that harness multiple antitumor mechanisms. As we advance toward clinical translation, Se@MI exemplifies how rational drug design can address complex biological challenges in cancer therapy.

## Data Availability

The datasets presented in this study can be found in online repositories. The names of the repository/repositories and accession number(s) can be found below: https://ngdc.cncb.ac.cn/gsa, CRA029356.
