# Supplementary material for: Selenium nanoparticle-delivered MDM2 inhibitor reactivates p53 and reprograms tumor immune microenvironment in colorectal cancer
Source: Front Immunol. 2025 Oct 31;16:1684611. doi: 10.3389/fimmu.2025.1684611 (PMC12615484; doi:10.3389/fimmu.2025.1684611)
Supplement: Supplementary file 1 [file Table1.docx]

**Supplementary Materials**

**Selenium nanoparticle-delivered MDM2 inhibitor reactivates p53 and reprograms tumor immune microenvironment in colorectal cancer**

**Weiming You** ^1,2^, **Jun Feng**^1,2^, **Litao Guo**^3^, **Jin Yan** ^1,2*^, **Siqi Yan** ^1,4*^

^1^Department of Hepatology, The Second Affiliated Hospital of Xi’an Jiaotong University, Xi’an, Shaanxi, China, ^2^Department of Tumor and Immunology in Precision Medical Institute, Western China Science and Technology Innovation Port, The Second Affiliated Hospital of Xi’an Jiaotong University, Xi’an, Shaanxi, China, ^4^Department of Critical Care Medicine, The First Affiliated Hospital of Xi’an Jiaotong University, Xi’an, Shaanxi, China, ^4^Institute for Stem Cell & Regenerative Medicine, The Second Affiliated Hospital of Xi’an Jiaotong University, Xi’an, Shaanxi, China

*** Correspondence:**

Jin Yan, yanjin19920602@xjtu.edu.cn

Siqi Yan, yansiqi92@xjtu.edu.cn

**Supplementary Figure**


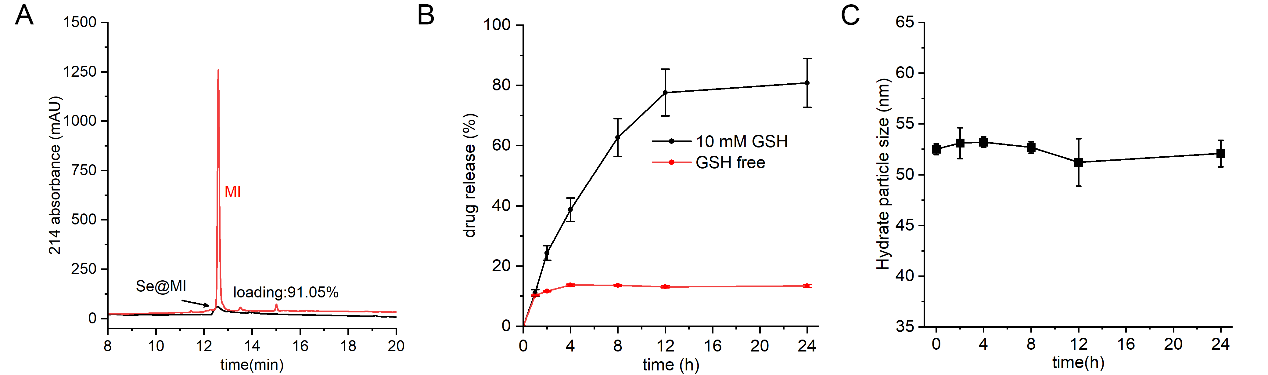


Figure S1. (A) RP-HPLC analysis for determination of MI loading efficiency in Se@MI nanoparticles. Detection wavelength: 214 nm. Time-dependent release profiles of MI from Se@MI nanoparticles in PBS (pH 7.4) under reductive conditions (10 mM GSH, black) and non-reductive conditions (GSH-free, red). (C) Hydrodynamic size stability of Se@MI monitored over 24 hours in PBS containing 20% FBS.

**
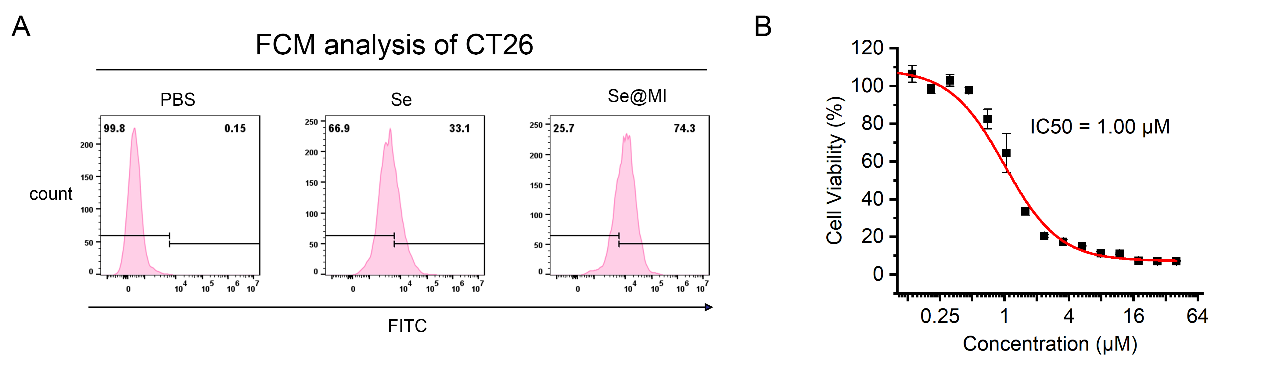
**

Figure S2. (A) Flow cytometry analysis of FITC-labeled nanoparticle uptake in CT26 cells after treatment with Se or Se@MI. (B) MTT assay performed to evaluate cell viability of CT26 cells treated with Se@MI at various concentrations.


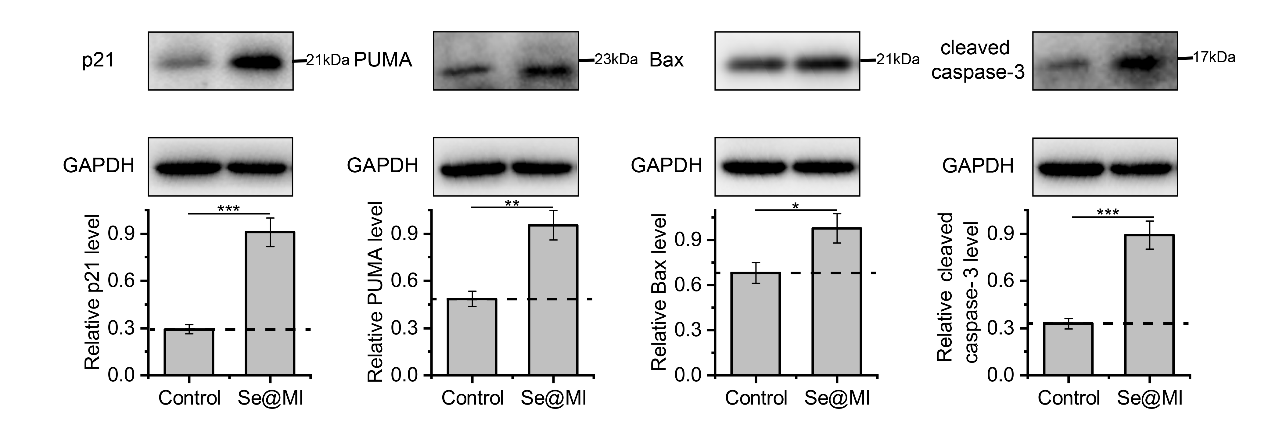


Figure S3. Western blot analysis of apoptosis-related proteins (p21, PUMA, Bax and cleaved caspase-3) in CT26 cells after treatment with Se@MI or Control (PBS). Data was presented as the mean ± SD. **P* < 0.05.***P* < 0.01; ****P* < 0.001.
